# Multi-view feature selection for identifying gene markers: a diversified biological data driven approach

**DOI:** 10.1186/s12859-020-03810-0

**Published:** 2020-12-30

**Authors:** Sudipta Acharya, Laizhong Cui, Yi Pan

**Affiliations:** 1 College of Computer Science and Software Engineering, Shenzhen University, Shenzhen, People’s Republic of China; 2 Department of Computer Science, Georgia State University, Atlanta, USA

**Keywords:** Gene selection, Sample classification, Gene ontology (GO), Protein–protein interaction network (PPIN), Multi-view learning, Multi-objective clustering, Gene similarity measures

## Abstract

**Background:**

In recent years, to investigate challenging bioinformatics problems, the utilization of multiple genomic and proteomic sources has become immensely popular among researchers. One such issue is feature or gene selection and identifying relevant and non-redundant marker genes from high dimensional gene expression data sets. In that context, designing an efficient feature selection algorithm exploiting knowledge from multiple potential biological resources may be an effective way to understand the spectrum of cancer or other diseases with applications in specific epidemiology for a particular population.

**Results:**

In the current article, we design the feature selection and marker gene detection as a multi-view multi-objective clustering problem. Regarding that, we propose an Unsupervised Multi-View Multi-Objective clustering-based gene selection approach called UMVMO-*select*. Three important resources of biological data (gene ontology, protein interaction data, protein sequence) along with gene expression values are collectively utilized to design two different views. UMVMO-*select* aims to reduce gene space without/minimally compromising the sample classification efficiency and determines relevant and non-redundant gene markers from three cancer gene expression benchmark data sets.

**Conclusion:**

A thorough comparative analysis has been performed with five clustering and nine existing feature selection methods with respect to several internal and external validity metrics. Obtained results reveal the supremacy of the proposed method. Reported results are also validated through a proper biological significance test and heatmap plotting.

## Background

The aim of analyzing microarray gene expression data is to expose meaningful biological information embedded within it, which in turn helps diagnose, prognosis, and determine the optimal treatment plan for any disease. Sample classification is one such gene expression data analysis technique that lets researchers identify categories of unknown diseased samples based on expression levels of genes. However, with the ever-increasing newly discovered biological data, handling the high-dimensional gene expression data sets has become a genuine problem which seeks a potential solution. The gene expression data set consists of expression values of thousands of genes, where only a small fraction of them shows a strong correlation with the targeted phenotypes. Therefore, as a popular solution of *‘Curse of dimensionality’* [1], in the past few years, various gene (or feature) selection methods [2–4] have been invented by researchers. Those methods aim to discard redundant genes from expression data sets and keep only a smaller subset of relevant genes that effectively participate in sample classification. These relevant and non-redundant genes are often recognized as disease-related genes or *gene-markers* [5–7], and they have a significant impact on genetic studies. Existing research indicates that genetic markers are highly involved in different cancer pathways; hence they can be useful for diagnosing and assessing drug efficacy and toxicity.

Owing to the nature of the problem, the biological data can be interpreted in different ways. In short, biological data are often multi-faceted or have multiple views. For example, characteristics of genes for a particular genome can be obtained from—(1) gene expression data in the form of expression levels; (2) Gene Ontology (GO) expressing semantic functionalities; (3) Protein–Protein-Interaction Network (PPIN) in the form of functional interactions between corresponding protein molecules; (4) protein sequence data in the form of encoded structural information of corresponding protein molecules and, etc. Although in recent years, several clustering-based feature-selection strategies [3, 8, 9] have been developed, but most of them follow single-view approaches, i.e., consider a single out of several available resources (mostly gene expression data [5] or GO [3]). Single-faceted or single-view clustering [10] algorithms refrain from considering several aspects of data-properties represented by other views. In contrast, considering multi-faceted biological data and treating them as multiple views while designing a clustering-based gene-selection method can reveal deep insights of functional relatedness between genes. Hence, multi-view clustering is believed to be more efficient for gene selection compared to single-view clustering techniques [11–13]. It is an exciting research challenge to combine multiple views or sources of the same set of instances to get a better clustering performance.

Recent advances of single-view clustering methods applied to complex biological data sets have proven the superiority of their multi-objective versions over single-objective counterparts [14, 15]. Hence, this fact must be applicable for multi-view clustering techniques as well. Motivated by this, in this current article, we propose an improved Unsupervised feature (gene) selection approach through a Multi-View Multi-Objective clustering method (called UMVMO-*select*). As the underlying optimization strategy of the proposed algorithm, the Archived Multi-Objective Simulated Annealing (AMOSA) [15] optimization technique is utilized.

Simulated annealing (SA) is a popular optimization algorithm that follows the principle of annealing metallurgy—a process involving heating and controlled cooling of a material to increase its crystals’ size and reduce their defects. To follow the annealing process of a metal, at first, the temperature is increased (up to $\texttt {T}_{\texttt {max}}$), then decreased very slowly up to a very low value ($\texttt {T}_{\texttt {min}}$). At each temperature, it ensures that the metal spends sufficient time. The searching strategy of SA also imitates this process. In [16], authors proved that if SA annealed sufficiently slowly, it converges to the global optimum. Because of the strong theory of SA, it has been applied in diverse areas [17, 18] where a single criterion is needed to be optimized. On the other side, there are very few works where multi-objective version of SA is proposed to solve the multi-objective optimization problems [19, 20]. Among the existing multi-objective SA algorithms [19, 20], AMOSA has been found to perform better because of its novel characteristics like constrained archive size, different forms of acceptance probability of new solution, incorporating the novel concept of the amount of domination in acceptance probability. According to existing literature [15, 21], the superiority of this optimization technique over existing optimization strategies has been proved experimentally. Therefore, in this current work, the choice of AMOSA to perform underneath our proposed UMVMO-*select* algorithm seems valid to identify optimal clustering solutions.

Our proposed feature selection approach considers different independent biological resources like gene expression data, GO, PPIN, and protein sequence data in a single framework, and cleverly develops two views from them. UMVMO-*select* then utilizes these views where, at each step, consensus clustering takes place to satisfy both views. Finally, it considers the encoded center genes of obtained optimal consensus gene clusters as the most informative and non-redundant genes. The selected genes further participate in the sample classification task. From the acquired reduced gene space, gene markers are chosen selectively. Please note that we use the term ‘features’ and ‘genes’ alternatively throughout the current manuscript.

### Related works and motivation

In the last few years, several efficient feature selection algorithms following different strategies have been proposed by researchers. Some existing pieces of literature relevant to this paper are discussed here briefly.

A single-objective genetic algorithm-based feature selection algorithm was proposed in the article [22]. Further, utilizing the obtained reduced feature space, kNN, and support vector machine (SVM) classifiers are used for sample classification. Similar to this work, in [8] also, the genetic algorithm has been employed for developing a multi-objective clustering-based feature selection approach. In [3], authors have proposed a single-objective clustering-based gene-selection algorithm without using expression data but utilizing GO’s biological information. However, their proposed method does not consider any other biological source except GO. Similarly, in [9], a gene-selection algorithm based on Clustering Large Applications based upon RANdomized Search (CLARANS) was proposed utilizing GO’s available biological knowledge. In the feature selection algorithms like Ranksum test [23] and *T*-test [24], the features are sorted according to their *p* values, and then the desired number of features from the list are chosen for validation. Though these methods successfully identify highly relevant features but fail to select non-redundant features. To overcome this shortcoming, authors of [25] have proposed a feature selection technique, namely MRMR (minimum redundancy–maximum relevance) feature selection, where genes are selected in such a way so that they are relevant as well as non-redundant to each other. The relevance of a feature is measured using mutual information between features, and redundancy is calculated using mutual information among features. With similar motivation, the authors of [5] proposed a feature selection method following a graph-theoretic approach, where a weighted dissimilarity graph was created based on the input gene expression data. In their developed graph, nodes represent genes and edges represent dissimilarity within connected genes. More the edge weight signifies more dissimilarity, and higher node weight indicates a higher relevance of the corresponding gene. Finally, they modeled the feature selection problem as a dense sub-graph finding problem and then solved through multi-objective binary particle swarm optimization (bPSO).

One common point among all literature, as mentioned earlier, is, all of them are single-view approaches. In recent years several multi-view clustering algorithms have been developed, but very few of them have been applied to biological research problems. For example, in [26], the authors’ proposed weighted multi-view clustering and feature selection technique is applied to real-life text and image data sets. Additionally, their proposed method follows a single-objective approach. Again, the authors of [27] proposed an unsupervised online multi-view feature selection algorithm for video or text data. A good survey on existing research on the development of multi-view clustering algorithms can be found in [13, 28]. On the problem of gene selection, a graph-theoretic multi-view clustering on gene expression data was proposed in [29]. Though their proposed algorithm is a multi-view approach, the views are developed based on expression data, and no other genomic/proteomic resources have been taken into account.

From the overall literature survey we did, it is clear that most of the existing gene selection algorithms have followed single-view approaches. Less attention has been provided on developing multi-view gene-selection strategies. Observing the above-mentioned limitation in the existing works, in the current paper, we propose an unsupervised multi-view multi-objective gene selection approach called UMVMO-*select*. The summary of our current work is presented as follows.

**Fig. 1:**
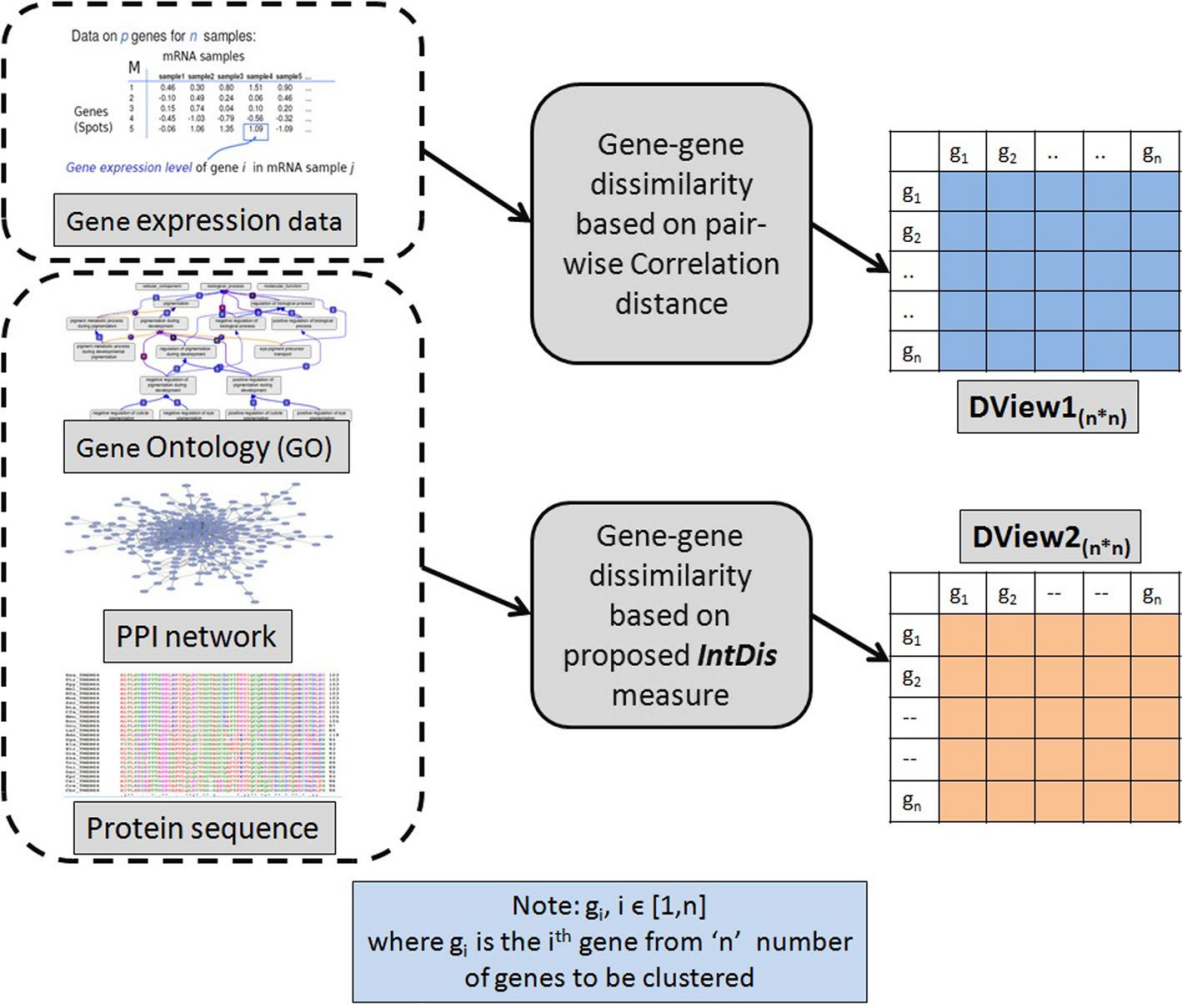
Generation of two views from 4 biological resources

- The primary contribution of this work is to propose a multi-objective clustering-based gene selection approach utilizing multi-view data that intuitively identifies relevant and non-redundant gene markers. The proposed method is unsupervised; hence, no labeled data has been utilized during feature-selection and gene-marker detection. As the underlying optimization strategy, AMOSA [15] has been used.
- An integrated gene dissimilarity measure based on GO, PPIN, and protein sequence called $\texttt {IntDis}$ has been proposed before defining views. Afterward, based on the correlation distance between gene expression vectors and proposed $\texttt {IntDis}$, we develop two different views in the form of two-dimensional gene-gene distance matrices (as shown in Fig. 1).
- UMVMO-*select* detects the number of optimal features or genes automatically.
- The concept of consensus partitioning is incorporated in the proposed method to satisfy both views.
- As objective functions, average Signal-to-Noise ratio (SNR) [30] (to measure gene relevance) and average correlation distance [31] (to measure gene non-redundancy) of consensus partitions for each clustering solution and agreement index (AI) [32] are optimized simultaneously.
- We conduct experiments on three benchmark cancer gene expression data sets; *Prostate cancer*, Diffuse large B-cell lymphomas (*DLBCL*), and *Child ALL*.^1^
- Finally, sample classification is carried out through multi-objective clustering for both original and reduced gene-spaces. A thorough comparative analysis with other existing single-objective/multi-objective single-view/multi-view feature selection algorithms has been carried out.

## Results

### Data sets and views

In the current work, all three chosen data sets are available in : www.biolab.si/supp/bi-cancer/projections/info/. The *Prostate cancer* data set contains gene expression values of both cancerous prostate tissues and normal prostate tissues. Originally this data set has 12,533 genes and 102 tissue samples. Among 102, the number of prostate tumor tissue samples is 52, and the rest 50 are normal tissue samples. On the other side, Diffuse large B-cell lymphomas (*DLBCL*) and follicular lymphomas (FL) are two B-cell lineage malignancies with very different clinical presentations, natural histories, and response to therapy. A total of 7070 number of genes and 77 number of samples are there. Out of 77 samples, 58 are of type DLBCL, and rest 19 are of type FL. *Child ALL* or GSE412 data set includes gene expression information on 110 childhood acute lymphoblastic leukemia samples over 8,280 genes. The samples are divided into two classes based on changes in gene expression before and after treatment, regardless of the type of treatment used. Out of 110, 50 samples are taken before any therapy, and rest 60 samples are taken after therapy.

First of all, the necessary pre-processing has been carried out on all data sets. The log transformation is performed on expression values, and after that, samples are normalized to variance 1 and mean 0. Once the data sets are pre-processed, next, for each of them, two views are generated. As mentioned before, views are represented in the form of dissimilarity matrix $\texttt {DView1}_{\texttt {n}\times \texttt {n}}$ and $\texttt {DView2}_{\texttt {n}\times \texttt {n}}$.

For generating *view-2*, the proposed $\texttt {IntDis}$ metric is calculated according to Eq. 8, and corresponding dissimilarity matrices $\texttt {DView2}_{\texttt {n}\times \texttt {n}}$ for all three data sets are generated. At first, the chosen data sets’ genes are mapped onto their corresponding significant GO-terms using a well known GO tool ; Gene Ontology Resource.^2^ Also, the full GO-tree is downloaded from the same source. For, *Prostate cancer* data set, out of 12,533 genes, 11,669 genes get mapped to significant GO-terms. The total number of mapped significant GO-terms (GO-terms having *p* value $\le$ 0.05) is 2300 (out of 2300, 1846 number of GO-terms under Biological Process (BP), 182 under Molecular Function(MF), and 272 under Cellular Component(CC) ontology). Similarly, for the *DLBCL* data set, out of 7070 genes, 5868 number of genes get mapped to corresponding GO-terms, and the number of significant GO-terms is 3444 (2821 under BP, 308 under MF, and 315 under CC). Also, for *Child ALL* data set, 6890 number of genes out of 8280 gets mapped to their corresponding GO-terms and the number of significant GO-terms is 2118 (1683 under BP, 162 under MF and 273 under CC). For all data sets, only mapped genes further take part in the gene selection process. The obtained annotation data and GO-tree are utilized for computing GO-based multi-factored similarity according to Eq. 3. To calculate the PPIN based similarity (according to Eq. 4) between genes of a data set, the full PPIN of *Homo Sapiens* or *H. Sapiens* organism is downloaded from HitPredict [33]—an open-access resource of experimentally determined protein–protein interaction data over several organisms. The corresponding protein UniProt IDs of mapped genes are downloaded from https://www.uniprot.org/. The protein IDs are used to retrieve their interacting proteins and associated statistics from downloaded *H. Sapiens* PPIN. For the last factor of the $\texttt {IntDis}$ measure, i.e., protein sequence-based similarity, at first bitscore matrix based on BLAST^3^ tool output is produced (as shown in Eq. 6). Please note that, during gene to protein ID mapping, due to alternative splicing [34], a single gene may get mapped into multiple protein IDs. In such cases, we choose one of these protein isoforms, which is also available in the BLAST tool database. If more than one protein isoforms are available in BLAST database, then any one of them is chosen arbitrarily.

Next, according to Eq. 7, protein sequence-based similarity between each pair of genes is measured. For those genes, which are not available in the BLAST database, the corresponding missing sequence-based similarity is replaced by the average of multi-factored similarity and PPI similarity. Finally, combining three measures according to Eq. 8, pairwise integrated distance measure between genes is calculated, and corresponding *view-2* or $\texttt {DView2}_{\texttt {n}\times \texttt {n}}$ is generated. For *Prostate cancer*, *DLBCL*, and *Child ALL* data sets, the dimensions of *view-2* or $\texttt {DView2}_{\texttt {n}\times \texttt {n}}$ are $(\texttt {11669} \times \texttt {11669})$, $(\texttt {5868} \times \texttt {5868})$ and $(\texttt {6890} \times \texttt {6890})$, respectively.

For *view-1*, pairwise correlation distance (according to Eq. 1) between each pair of genes for all three preprocessed data sets is calculated. To make the dimension of *view-1* dissimilarity matrix $\texttt {DView1}_{\texttt {n}\times \texttt {n}}$ the same with *view-2*, we do not consider the chosen data sets’ unmapped genes. For *Prostate cancer*, *DLBCL*, and *Child ALL* data sets, the dimensions of *view-1* or $\texttt {DView1}_{\texttt {n}\times \texttt {n}}$ are $(\texttt {11669} \times \texttt {11669})$, $(\texttt {5868} \times \texttt {5868})$ and $(\texttt {6890} \times \texttt {6890})$, respectively.

### Input parameters of UMVMO-*select*

As the underlying optimization strategy of the proposed UMVMO-*select* follows AMOSA [15]; therefore, some parameters related to this optimization algorithm are needed to be initialized with certain values as follows.

$\texttt {T}_{\texttt {min}} = 0.0001$, $\texttt {T}_{\texttt {max}} = 100$, $\alpha = 0.9$, $\texttt {HL} = 50$, $\texttt {SL} = 100$ and $\texttt {TotalIter} = 100$. $\texttt {K}_{\texttt {min}}$ or the minimum number of clusters = 2 and $\texttt {K}_{\texttt {max}}$ or the maximum number of clusters = $\sqrt{\texttt {n}}$, where $\texttt {n}$ number of genes/features to be clustered.

The above-mentioned parameter values are determined after conducting a thorough sensitivity study. According to [15], the initial value of the temperature or $\texttt {T}_{\texttt {max}}$ should be chosen high to allow the SA to perform a random walk over the landscape. The geometrical cooling schedule $\alpha$ is chosen in the range between 0.5 and 0.99 accordingly. We vary the value of $\alpha$ between this range by keeping other parameters constant. Finally, the value of $\alpha$ for which we got the best Silhouette measure [35] for the produced gene clustering solution is chosen as the cooling rate. Another important factor, i.e., the number of iterations per temperature or $\texttt {TotalIter}$, should be chosen so that the system is sufficiently close to the stationary distribution at that temperature. We choose the value of $\texttt {TotalIter} = 100$. By further increasing the value of $\texttt {TotalIter}$, the Silhouette value of the resulting gene clustering solution did not improve. So we fixed it to $\texttt {TotalIter} = 100$. To get consistent and standard solutions for all the chosen data sets, we consider the above-mentioned parameters setting.

**Table 1:** Comparative Silhouette index values for obtained gene-clusters by proposed as well as other single-view clustering methods

Data sets	# of clusters	UMVMO-select	UMC-view-1	UMC-view-2	PAM-view-1	PAM-view-2	Acharya et al. [3]
*Prostate*	45	0.427	0.403	0.414	0.392	0.399	0.397
*DLBCL*	37	0.432	0.427	0.429	0.409	0.421	0.412
*Child ALL*	33	0.443	0.405	0.418	0.384	0.399	0.39

### External and internal validity measures

The comparative analysis has been performed during different stages of the current experiment. For that purpose, three widely used internal cluster validity measures viz. Silhouette index [35], DB index [36], and Dunn index [37] have been utilized, and comparative results for them are reported in this article. Higher values of the Silhouette and Dunn index represent a better clustering solution. On the other side, a lower value of the DB index indicates a better clustering solution. Also, to compare sample classification outcomes with true sample classes, four well-known external validity indices: F-score, Sensitivity, Specificity, and Classification accuracy (CA) are reported. Higher values of these chosen external indices represent better classification outcomes.

**Table 2:** Comparative analysis of obtained sample classification outputs applied on original and reduced gene space

Data sets	# of genes (features)	# of samples	Silhouette	DB	Dunn
*Prostate*	12,555 (original)	102	0.352	0.835	0.779
	45 (reduced)		0.3921	0.8243	0.791
*DLBCL*	7070 (original)	77	0.357	0.776	0.716
	37 (reduced)		0.3772	0.779	0.725
*Child ALL*	8280 (original)	110	0.322	0.723	0.713
	33 (reduced)		0.339	0.721	0.7131

**Table 3:** Comparative analysis of proposed gene selection algorithm UMVMO-*select* with other existing gene selection methods with respect to sample classification outputs

Algorithm	Sensitivity			Specificity			F-score			CA		
	*Prostate*	*DLBCL*	*Child ALL*	*Prostate*	*DLBCL*	*CHild ALL*	*Prostate*	*DLBCL*	*Child ALL*	*Prostate*	*DLBCL*	*Child ALL*
UMVMO-*select*	0.9038	**0**.**948**	**0**.**8**	**0**.**92**	0.842	0.833	**0**.**9125**	**0**.**948**	**0**.**8**	**0**.**91176**	**0**.**922**	**0**.**8181**
Acharya et al. [3]	0.8846	0.9137	0.72	0.92	0.7894	0.8166	0.9019	0.921	0.742	0.9019	0.8831	0.7727
Graph-MPSO [5]	0.8962	0.9111	0.752	0.9	**0**.**9207**	0.8233	0.9002	0.8428	0.7671	0.898	0.9184	0.7909
Graph-SingleObjective (Correlation)[5]	0.8221	0.6389	0.71	0.855	0.8966	0.8042	0.8382	0.639	0.7295	0.8382	0.8355	0.7614
Graph-SingleObjective (SNR)[5]	0.8701	0.8333	0.64	0.865	0.8707	0.8442	0.8704	0.7434	0.7079	0.8676	0.8618	0.7568
*T*-test [24]	0.7778	0.7284	0.4640	0.8244	0.9119	0.68	0.8336	0.7052	0.5184	0.8497	0.8486	0.5964
RankSum test[23]	0.8547	0.7654	0.4640	0.8375	0.8945	0.87	0.8522	0.7327	0.5506	0.8768	0.8621	0.6855
MRMR[25]	**0**.**9176**	0.8889	0.7486	0.8686	0.9163	**0**.**8762**	0.8970	0.8244	0.7896	0.8936	0.9098	0.7782
Acharya et al.[15]	0.865	0.8965	0.68	0.88	0.7368	0.8	0.8735	0.904	0.708	0.8725	0.8571	0.7454
Saha et al. [14]	0.8461	0.8793	0.64	0.86	0.6842	0.7833	0.8543	0.8869	0.6736	0.8529	0.8311	0.7181

## Discussion

We execute UMVMO-*select* ‘$\texttt {t}$’ times to perform gene clustering for gene selection and sample clustering with reduced/original gene space for each of the chosen data sets. The different ‘$\texttt {t}$’ values for different data sets are mainly decided based on the saturation level in the identified set of gene-markers. The details are discussed in a later section. All seven validity measures (three of them internal and rests four are external indices) are computed for each run. Finally, the average of obtained ‘$\texttt {t}$’ sets of validity measures is reported in Tables 1, 2, and 3.

We compare the performance of our proposed method for gene clustering with five other alternative clustering techniques, which are single-view versions of the proposed algorithm (unsupervised multi-objective clustering (UMC) with *view-1* and *view-2*), the single-view single-objective clustering method developed in our previous work on gene selection [3], and PAM clustering utilizing our developed views.

Also, the efficiency of selected genes by our algorithm UMVMO-*select* in sample classification is compared with nine other existing gene selection and sample classification methods. These are the approach of Acharya et al. [3], Wilcoxom RankSum test [23], *T*-test [24], graph-theoretic multi-objective PSO [5] and it’s two single-objective versions, MRMR feature selection [25], the approach of Acharya et al. [15], and feature weighing based approach of Saha et al. [14].

### Results for *Prostate cancer* data set

After performing our proposed multi-view multi-objective clustering on the *Prostate cancer* data set, before selecting candidate genes, the ensembled clustering solution’s quality is measured through the Silhouette index and compared with other single-objective/single-view clustering techniques. We execute our algorithm 5 times, so for this data set $\texttt {t=5}$ and hence, 5 ensembled clustering solutions are obtained. The average Silhouette index values for all 5 solutions are reported in Table 1. The number of clusters in the best clustering solution (among 5 runs) with respect to Silhouette value is reported in the table. From the reported results, it is evident that our proposed algorithm outperforms reported single-view clustering algorithms with respect to obtained Silhouette measure. It supports the superiority of multi-view clustering over single-view approaches. To verify the biological significance of all five ensembled clusters by UMVMO-*select*, we perform GO enrichment analysis on their gene-clusters using the GO tool. The outcome of the biological significance test on random two clusters of the best ensembled solution (the solution with best Silhouette value) has been tabulated in Table 4. In the table for each of the significant GO-terms, the percentage of genes from the obtained cluster and full genome set in GO tool sharing that term is reported. It is quite evident from Table 4 that a higher percentage of genes from the obtained ensembled clusters mapped into the corresponding GO-term compared to the full genome set. This indicates that genes of the same obtained clusters are more involved in similar biological processes compared to remaining genes in the genome. We validate all of 5 ensembled clustering solutions through the GO enrichment test.

Next, the centers of the best ensembled solution (with respect to Silhouette index) are extracted. Hence, the reduced set of most relevant and non-redundant genes ($\texttt {Cand}$) is formed. Afterward, we perform the single-view AMOSA-based clustering proposed by Acharya et al. [3] on samples for classification utilizing both original and reduced gene-space. The obtained results on sample clustering for the *Prostate cancer* data set are reported in Table 2. We can see, compared to the original dimension (12555 genes), our proposed algorithm has reduced the number of genes to a great extent (45 genes). If we compare the quality of obtained sample clusters, it is clear from Table 2 that, sample clustering solution with reduced gene space is better in quality according to reported Silhouette, DB, and Dunn index (0.3921, 0.8243, 0.791).

We also compare obtained classes of samples with their original class levels, and the performance has been evaluated through four external validity measures, which are reported in Table 3. For *Prostate cancer* data set, it can be observed that, with respect to Specificity, F-score, and Classification accuracy, our proposed UMVMO-*select* performs best (0.92, 0.9125, 0.91176) among all nine feature selection techniques. Regarding sensitivity, our algorithm outperforms all other reported methods except MRMR [25].

**Table 4:** Biological significance test outcome for two obtained random clusters by UMVMO-*select* on *Prostate cancer* data set (out of 45 clusters)

Cluster	GO term	Genome %	Cluster %
Cluster 1	GO:0051716	31.9	43.7
289	Cellular response to stimulus		
	GO:0019222	31.8	43.4
	Regulation of metabolic process		
	GO:0031323	29.6	40
	Regulation of cellular metabolic process		
	GO:0060255	29.3	39.8
	Regulation of macromolecule metabolic process		
Cluster 2	GO:0031326	20	26.5
242	Regulation of cellular biosynthetic process		
	GO:0051674	27.4	37.5
	Localization of cell		
	GO:0048519	25.4	36.9
	Negative regulation of biological process		
	GO:0051234	22.3	31.2
	Establishment of localization

**Table 5:** Biological significance test outcome for two obtained random clusters by UMVMO-*select* on *DLBCL* data set (out of 37 clusters)

Cluster	GO term	Genome %	Cluster %
Cluster 1	GO:0050896	40.4	61.2
211	Response to stimulus		
	GO:0044260	24.2	50
	Cellular macromolecule metabolic process		
	GO:0016043	26.8	63.2
	Cellular component organization		
	GO:0048518	29.5	50.9
	Positive regulation of biological process		
Cluster 2	GO:0044238	35.7	58.2
177	Primary metabolic process		
	GO:1901564	25.3	37.9
	Organonitrogen compound metabolic process		
	GO:0071704	37.5	55.8
	Organic substance metabolic process		
	GO:0048856	25.8	40.8
	Anatomical structure development

**Table 6:** Biological significance test outcome for two obtained random clusters by UMVMO-*select* on *Child ALL* data set (out of 33 clusters)

Cluster	GO term	Genome %	Cluster %
Cluster 1	GO:0007399	11.4	19.27
187	Nervous system development		
	GO:0071840	21.68	40.66
	Cellular component organization or biogenesis		
	GO:0007165	24.62	36.44
	Signal transduction		
	GO:1901564	25.41	39.45
	Organonitrogen compound metabolic process		
Cluster 2	GO:0048856	26.14	35.9
209	Anatomical structure development		
	GO:0071840	27.68	42.5
	Cellular component organization or biogenesis		
	GO:0006139	13.49	27.34
	Nucleobase-containing compound metabolic process		
	GO:0051239	15.28	34.58
	Regulation of multicellular organismal process		

### Results for *DLBCL* data set

Similar to *Prostate cancer*, the obtained results for the *DLBCL* data set are also analyzed thoroughly. Here also we choose $\texttt {t=5}$. From the reported values of the Silhouette index in Table 1, it is evident that here also our method outperforms other single-view clustering algorithms to identify quality clusters of functionally similar genes. The obtained gene clusters are validated biologically through GO enrichment analysis, and the obtained test outcome is reported in Table 5 for random two clusters from best ensembled solution. Like previous data set here also we observe the reported clusters are biologically significant.

In Table 2, the reported results show that according to the obtained Silhouette and Dunn index, our method with reduced gene space (37 genes) produces better sample clusters than the original gene space (0.3772, 0.725). However, with respect to the DB index, sample clusters’ quality slightly degrades for reduced (0.779) than the original gene space. Also, if we compare the reported results of Table 3, it is clear that with respect to Sensitivity, F-score, and Classification accuracy, our method performs much better than other existing nine feature selection techniques (0.948, 0.948, 0.922). However, with respect to Specificity, we observe that the existing graph-theoretic MPSO based feature selection technique [5] performs best among all.

### Results for *Child ALL* data set

The outcome of the comparative study for *Child ALL* is also reported in the current manuscript. Here we choose the value of ‘$\texttt {t}$’ as $\texttt {t=6}$. Table 1 shows that, like the other two chosen data sets, the quality of the obtained gene clusters by the proposed approach has been proven to be better compared to other single-view approaches. The obtained 6 ensembled gene clustering solutions are also biologically validated through GO enrichment analysis. The significance test outcome for random two clusters from the best solution for this data set is shown in Table 6.

From the results reported in Table 2, it can be observed that the number of genes in the reduced set is 33, which is much lesser than the original dimension (8280 genes) of the *Child ALL* data set. Also, with the reduced gene space, the Silhouette and DB index values corresponding to the sample clustering solution are better (0.339, 0.721) than the original gene space. The Dunn index value is found almost the same (0.7131) for both original and reduced gene space. The comparative sample classification outcome of this data set is also shown in Table 3. Similar to the *DLBCL* data set, here also with respect to Sensitivity, F-score, and Classification accuracy, our method performs better than other existing nine feature selection techniques (0.8, 0.8, 0.8181). For Specificity, we observed MRMR [25] performed best among all.

**Table 7:** Comparative analysis of proposed gene selection algorithm UMVMO-*select* over different combination of objective functions

*Objective* function	CA		
	*Prostate*	*DLBCL*	*Child ALL*
SNR	0.8333	0.792	0.727
Corr. dist.	0.754	0.7532	0.68
AI	0.784	0.779	0.709
SNR+Corr. dist.	0.901	0.8701	0.79
Corr. dist. + AI	0.872	0.831	0.763
SNR + AI	0.892	0.8441	0.781
**SNR + Corr. dist. + AI**	**0**.**91176**	**0**.**922**	**0**.**8181**

### Effect of different *‘omic’* data sources and objective functions on sample classification accuracy

To investigate the effect of different *‘omic’* data sources and objective measures on proposed UMVMO-*select*, we perform experiments with different sets of views and objective functions. Table 7 reports results about the effect of different combinations of objective functions on our proposed method. Note that we kept the views selection as same as our original algorithm. Only objective functions are varied. The last row (marked as **bold**) of the table is the sample classification accuracy while considered all three objective functions as the original algorithm does. It is evident from the reported results that the combination of three objective functions in UMVMO-*select* made it performing best compared to lesser objectives selection.

Similarly, in Table 8, we vary the chosen *‘omic’* data sources in produced views and reported the obtained sample classification accuracy. As *view-1*, we always select gene expression (GE) data. For *view-2*, we choose different combinations of data sources. Please note, here we have choose all three objective functions as our original algorithm does. The last row (marked as **bold**) of the table reports the results considering all data sources. According to reported results in this table, the proposed approach’s superiority proves the importance of considering multiple *‘omic’* sources to design views.

**Table 8:** Comparative analysis of proposed gene selection algorithm UMVMO-*select* over different combination of views

*View-1*	*View-2*	CA		
		*Prostate*	*DLBCL*	*Child ALL*
GE	GO	0.7941	0.7922	0.736
	PPIN	0.8039	0.8051	0.745
	BLAST	0.6862	0.7532	0.7
	GO+PPIN	0.8725	0.8971	0.809
	PPIN+BLAST	0.8333	0.8571	0.781
	GO+BLAST	0.8235	0.8181	0.772
	**GO+PPIN+BLAST**	**0**.**91176**	**0**.**922**	**0**.**8181**

### Retrieved marker genes

As we have seen before, for each of the chosen data sets, ‘$\texttt {t}$’ sets of features/reduced gene-space (by selecting gene-centers) are obtained. The common centers over ‘$\texttt {t}$’ number of runs for all chosen data sets are tagged as marker genes. For the *Prostate cancer* data set, the reason behind choosing $\texttt {t=5}$ is after further increasing the ‘$\texttt {t}$’ (runs), we did not notice any change in the set of the obtained gene markers. For the *DLBCL* data set, we observe the set of gene markers saturates after $\texttt {t=4}$. However, for $\texttt {t=5}$, the average Silhouette, DB, and Dunn indices values corresponding to the obtained sample clustering are found better compared to $\texttt {t=4}$. Therefore, for this data set, we report all experimental results for $\texttt {t=5}$. For *Child ALL* data set, we choose $\texttt {t=6}$ for reporting comparative results in Tables 1, 2, and 3 and for gene marker identification for the same reason. The symbol, ID, description, and regulation status of identified gene markers for all data sets are provided in Table 9. To study the biological relevance of the obtained gene markers, many of those have been validated to be associated with the respective cancer classes in the different existing literature. For example, in the *Prostate cancer* data set, gene 41288*_at* (CALM1) and 32243*_g_at* (CRYAB) have been also reported in [38].

**Table 9:** Identified cancer gene markers by proposed method for all three data sets

Data set	Gene ID	Gene name	Description	Regulation mode
*Prostate*	32243*_g_at*	CRYAB	Crystallin, alpha B	Up
*cancer*	41288*_at*	CALM1	Calmodulin 1	Up
	37639*_at*	HPN	Hepsin	Up
	41504*_s_at*	MAF	v-maf musculoaponeurotic fibrosarcoma oncogene homolog	Up
	40435*_at*	SLC25A6	Solute carrier family 25, member 6	Down
	33614*_at*	RPL18A, RPL18AP3	Ribosomal protein L18a, L18a pseudogene 3	Down
	1657*_at*	PTPRR	Protein tyrosine phosphatase receptor type R	Down
*DLBCL*	X02152*_at*	LDHA	Lactate dehydrogenase	Up
	M25753*_at*	CCNB1	Cyclin B1	Up
	U59309*_at*	FH	Fumarate hydratase, mitochondrial precursor	Up
	M16336*_s_at*	ENO1	Enolase 1	Down
*Child ALL*	32659*_at*	EIF2B4	Translation initiation factor eIF-2B subunit delta	Up
	39221*_at*	LILRB2	Leukocyte immunoglobulin-like receptor subfamily B member 2	Up
	41117*_s_at*	SLC9A3R2	Solute carrier family 9, isoform 3 regulator 2	Down
	33069*_f_at*	UGT2B15	UDP glucuronosy1transferase 2 family, polypeptide B15	Down
	37226*_at*	BNIP1	BCL2/adenovirus E1B 19 KDa interacting protein 1	Down
	34757*_at*	PARP2	Poly (ADP-ribose) polymerase 2	Down

**Fig. 2:**
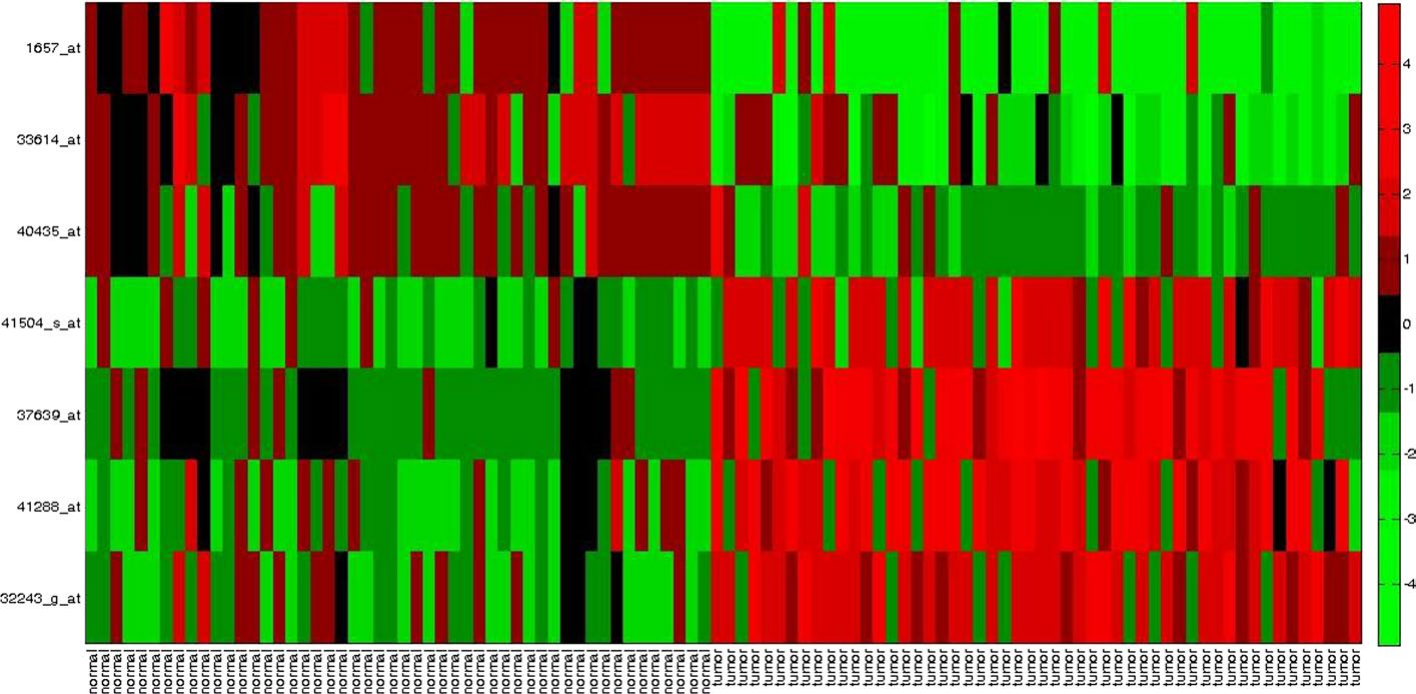
Heatmap for obtained gene markers for *Prostate cancer* data set

**Fig. 3:**
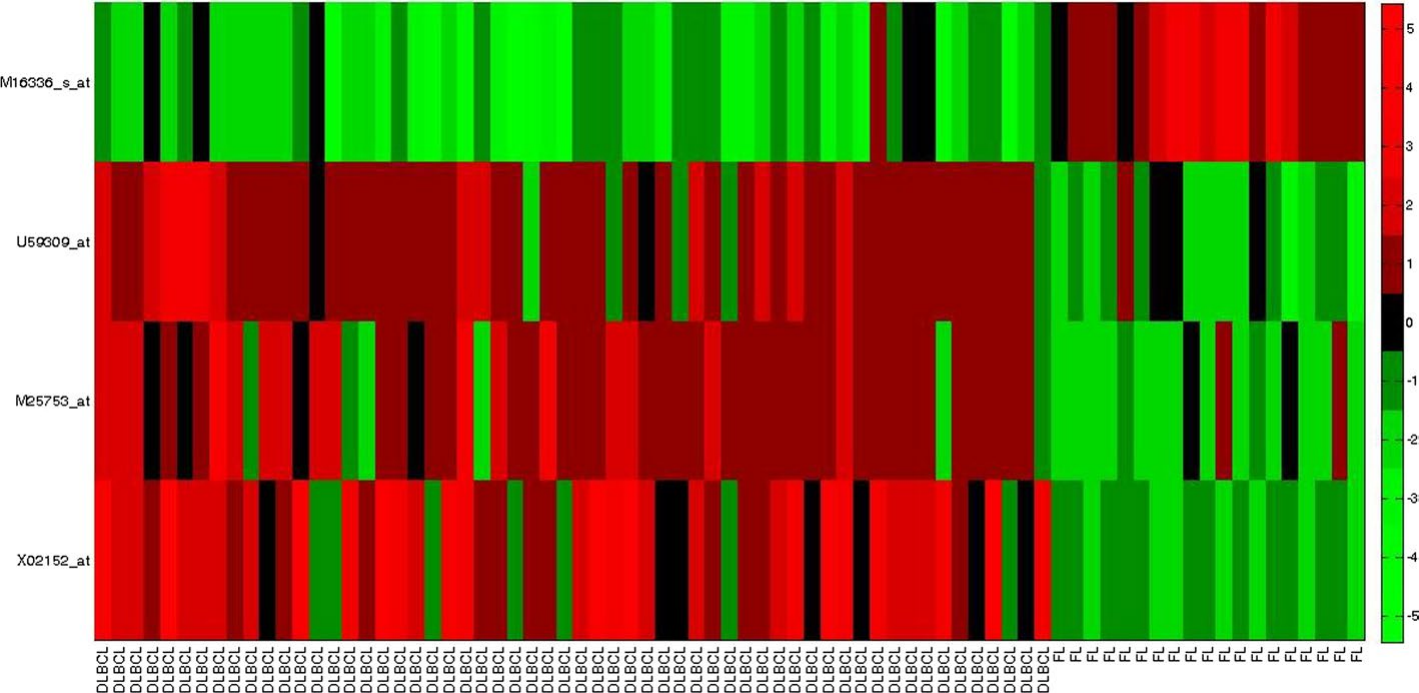
Heatmap for obtained gene markers for *DLBCL* data set

**Fig. 4:**
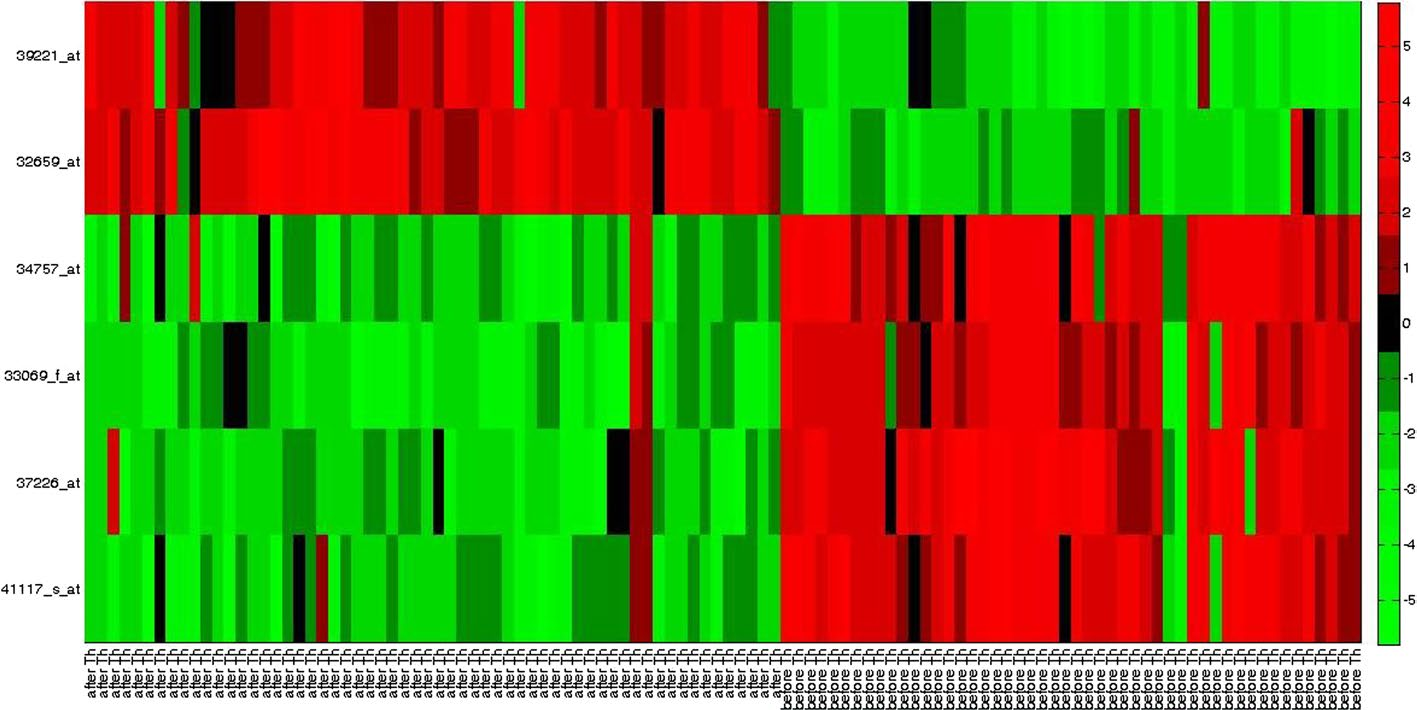
Heatmap for obtained gene markers for *Child ALL* data set

Again, gene 40435*_at* (SLC25A6) and 33614*_at* (RPL18A, RPL18AP3) in [5], and 37639*_at* (HPN) and 41504*_s_at* (MAF) have been reported in [39]. Similarly, for the *DLBCL* data set we observe, X02152*_at* (LDHA) and M25753*_at* (CCNB1) have been identified by [40], U59309*_at* (FH) has been reported in [5]. For the *Child ALL* data set, 41117*_s_at* (SLC9A3R2) and 33069*_f_at* (UGT2B15) have been reported in [41]. Also, 37226*_at* (BNIP1) and 34757*_at* (PARP2) were identified by [5]. In Figs. 2, 3, and 4, we have plotted the heat maps for obtained gene markers for all three data sets. The heat map is used to visually illustrate that the identified gene-markers are differentially expressed, i.e., actually contain the essential property of an ideal gene-marker.

Every row of a heat map represents each of the chosen gene-markers from the corresponding data set. Each cell of the heat map represents the expression level of a gene for the corresponding sample. The *red* cell indicates a high expression value, whereas the *green* cell represents a lower value of an expression. The *black* cell indicates an average expression value. To be differentially expressed, a marker gene is needed to be either up-regulated (high expression value) or down-regulated (low expression value) for each tissue sample of the respective tumor class. In Fig. 2, we can see for the *Prostate cancer* data set, the obtained marker genes are either up-regulated or down-regulated for most of the samples over both classes. For example, gene 32243*_g_at* (CRYAB), 41288*_at* (CALM1), 37639*_at* (HPN), and 41504*_s_at* (MAF) are up-regulated (high expression values in tumor class and low expression values in normal sample class). On the counterpart, 40435*_at* (SLC25A6), 33614*_at* (RPL18A, RPL18AP3), and 1657*_at* (PTPRR) are down-regulated (high expression values in normal class and low expression values in tumor class). Similarly, if we study the heatmap of *DLBCL* data set in Fig. 3, we can see gene X02152*_at* (LDHA), M25753*_at* (CCNB1), and M16336*_s_at* (ENO1) are up-regulated (high expression in DLBCL and low expression in FL) and U59309*_at* (FH) is down-regulated (high expression for FL and low expression for DLBCL). For *Child ALL* data set, as we can observe from Fig. 4 that, gene marker 41117*_s_at*, 33069*_f_at*, 37226*_at*, 34757*_at* are down-regulated (high expression values in ‘before therapy’ (before Th) class and low expression values in ‘after therapy’ (after Th) class). On the other side, gene marker 32659*_at* and 39221*_at* are up-regulated (low expression values in ‘before therapy’ (before Th) class and high expression values in ‘after therapy’ (after Th) class).

## Conclusion

In the current article, we propose an unsupervised multi-view multi-objective gene selection approach called UMVMO-*select*, which intuitively identifies gene markers from the chosen cancer gene expression data sets. Multiple *‘omic’* data sources like gene expression, GO, PPIN, and protein sequence are amalgamated to build two views. Experiments are carried out on three cancer gene expression data sets; *Prostate cancer*, *DLBCL*, and *Child ALL*. From the thorough comparative analysis with existing feature selection algorithms and several validation tests, we observe that our proposed method reduces the original gene space significantly and improves the sample classification accuracy. From the obtained experimental outcomes, we also observed that incorporating more relevant data sources in designing views increases the overall efficiency of the multi-view clustering approach. Therefore, in the future, more views can be identified based on other genomic/proteomic resources [42–44], and comparative experiments can be performed to observe the effect of increased views on sample classification accuracy. Also, apart from gene selection, our proposed multi-view based clustering approach can be applied to solve other interesting bio-informatics problem like hub-protein detection [45], essential protein identification, etc. The authors are currently working in that direction.

## Methods

In this section, at first, we describe the development mechanism of both views in detail. Next, we elaborate on different steps of the proposed UMVMO-*select*.

### Two views for UMVMO-*select*

Two gene-gene dissimilarity networks developed utilizing multiple *‘omic’* data sources are treated as two different views. The first view is the gene dissimilarity network based on pair-wise correlation distance [31] between expression vectors of genes. For the second view, gene dissimilarity network is created utilizing our newly proposed integrated gene-gene dissimilarity measure $\texttt {IntDis}$. The proposed measure incorporates biological properties of GO, corresponding organism’s PPIN, and protein sequence structure. Figure 1 illustrates the formation steps of both views. Both developed views are essential. The first view represents dissimilarity between genes based on their sample-specific expression levels. In contrast, the other view signifies the semantic and functional dissimilarity between genes according to GO, PPIN, and protein sequence cumulatively, which is not specific to samples but captures a global functional relatedness among genes.

Mathematically, suppose $\texttt {n} = \#$ of genes and $\texttt {d}= \#$ of samples in a given gene expression data set. The original expression data is represented as $\texttt {G}_{\texttt {org}}[\texttt {n}][\texttt {d}]$. $g_i$ represents $i^{th}$ gene of data set where $i \in \texttt {n}$. The dissimilarity network for *view-1* is represented as two-dimensional distance matrix $\texttt {DView1}_{\texttt {n}\times \texttt {n}}$ of dimension $\texttt {n} \times \texttt {n}$. The dissimilarity between expression vectors of each pair of genes is calculated using the correlation coefficient. The correlation coefficient $\varphi$ and correlation distance between two random variables *a* and *b* can be defined as follows [31].

1 $$\begin{aligned} \varphi (a,b)= & {} \frac{\texttt {cov}(a,b)}{\sqrt{\texttt {var}(a)\texttt {var}(b)}} \nonumber \\ Correlation\,distance= & {} (1 - \vert \varphi (a,b)\vert ) \end{aligned}$$

Here $\texttt {cov()}$ denotes the covariance between variables and $\texttt {var()}$ denotes the variance of a variable. If variables *a* and *b* are correlated to each other, i.e., exact linear dependency exists, then $\varphi (a,b) = 1$ or $-1$ and if uncorrelated, then $\varphi (a,b)=0$. Therefore, ($1- \vert \varphi (a,b)\vert$) represents the dissimilarity between variables *a* and *b* or correlation distance. All entries of the $\texttt {DView1}_{\texttt {n}\times \texttt {n}}$ matrix are calculated according to Eq. 1.

For building the second view, at first, we propose an integrated gene-gene dissimilarity measure $\texttt {IntDis}$ combining biological knowledge obtained from GO, PPIN, and protein sequence.

To design $\texttt {IntDis}$, three key similarity measures based on chosen genomic/proteomic resources are,

1. Multi-factored protein–protein similarity based on GO annotation data ([21])
2. Functional similarity between proteins based on the confidence of association in PPIN ([46])
3. Protein sequence-based similarity utilizing Basic Local Alignment Search Tool (BLAST) based bitscore ([47])

The multi-factored gene similarity measure [21] captures functional and semantic relatedness between genes based on different information-theoretic, topological and structural properties of GO-terms and GO-tree.

Let $A_i$ and $A_j$ represent sets of annotated GO-terms by genes $g_i$ and $g_j$ respectively from the set of genes of original data set $\texttt {G}_{\texttt {org}}[][]$. According to [21], the multi-factored semantic similarity between two GO-terms $got_i$ and $got_j$ is as follows.

2 $$\begin{aligned} \texttt {Multi}\hbox {-}{}\texttt{sim}(got_i,got_j) = \frac{arctan[\texttt {Y}]}{\pi /2} \end{aligned}$$

Here $$\texttt {Y} = \texttt {sim}_{\texttt {Lin}}(got_i,got_j)+ \texttt {sim}_{\texttt {Shen}}(got_i,got_j)+ \texttt {sim}_{\texttt {norm}\hbox {-} \texttt {struct}_{\texttt {depth}}}(got_i,got_j)$$.

$\texttt {sim}_{\texttt {Lin}}(got_i,got_j)$, $\texttt {sim}_{\texttt {Shen}}(got_i,got_j)$ and $\texttt {sim}_{\texttt {norm}\hbox {-} \texttt {struct}_{\texttt {depth}}}(got_i,got_j)$ represents GO-terms similarity based on Lin’s semantic similarity measure [48], Shen’s similarity measure [49], and normalized structure-based semantic similarity [21] respectively.

Utilizing Eq. 2, the multi-factored semantic similarity between gene $g_i$ and $g_j$ is as follows.

3 $$\begin{aligned} \texttt {Multi}\hbox {-}{}\texttt{SIM}(g_i,g_j) = \frac{\frac{1}{m \times n}\sum \limits _{got_k \in A_i, got_p \in A_j} \texttt {Multi}\hbox {-}{}\texttt{sim}(got_k,got_p) + \texttt {sim}_{\texttt {NTO}}(g_i,g_j)}{2} \end{aligned}$$

Here $sim_{NTO}(g_i,g_j)$ is normalized term overlap-based similarity measure [21] and $\texttt {m} = \vert A_i \vert$ and $\texttt {n} = \vert A_j \vert$. Also, $\texttt {Multi-SIM}(g_i,g_j)\in [0,1]$.

Again let, the corresponding gene-product or protein of gene $g_i$ is denoted by $p_i$. $N_i$ denotes the set of interactive proteins of $p_i$ in its corresponding PPIN. $w_{ij}$ indicates the confidence score or weight of the edge between interacting protein $p_j \in N_j$ and $p_i$. Let $N_{ij}$ is the set of proteins that are interactive neighbors of both protein $p_i$ and $p_j$, i.e., $N_{ij}$ = $N_i \cap N_j$. $\tilde{N_i}$ = $N_i \backslash N_{ij}$ indicates the set of proteins, which are interactive neighbors of protein $p_i$ but not of protein $p_j$. Hence, based on the confidence (here weight) of association within PPIN, the functional similarity between gene $g_i$ and $g_j$ in PPIN [46] is defined as follows.

4 $$\begin{aligned} \texttt {PPI}\hbox {-}{}\texttt{SIM}(g_i, g_j) = \frac{\sum \limits _{{g_k} \in N_{ij}} min\lbrace w_{ik},w_{jk} \rbrace }{\sum \limits _{g_k \in \tilde{N_i}} w _{ik} + \sum \limits _{{g_k} \in N_{ij}} max\lbrace w_{ik},w_{jk} \rbrace + \sum \limits _{g_k \in \tilde{N_j}} w _{jk}} \end{aligned}$$

Also, $$\texttt {PPI-SIM}(g_i, g_j) \in [0,1]$$.

Apart from GO and PPIN, the proposed $\texttt {IntDis}$ measures protein similarity based on protein sequence structure. For this purpose, BLAST^4^ is utilized to measure sequence alignment similarity between two protein molecules. The bit score represents a normalized raw sequence alignment score, which is expressed in bits. It represents how proper the alignment is; the higher the score, the better the alignment. As the BLAST output is not symmetric, the sequence similarity between gene $g_i$ and $g_j$ is obtained by taking average [47] of two BLAST results as follows.

5 $$\begin{aligned} \texttt {sim}_{\texttt {BLAST}} = log_{10} \frac{\texttt {Bitscore}(g_i,g_j) + \texttt {Bitscore}(g_j,g_i)}{2} \end{aligned}$$

According to Eq. 5, between each pair of protein molecules, we calculate the sequence similarity score, and hence sequence alignment based similarity matrix $\texttt {bit}\hbox {-} \texttt {matrix}[][]$ is formed. Next, the entries of the matrix are normalized within 0 and 1 as follows.

6 $$\begin{aligned} \texttt {Bitscore}_{\texttt {norm}}= \frac{\texttt {Bitscore} - minimum(\texttt {Bitscore})}{maximum(\texttt {Bitscore}) - minimum(\texttt {Bitscore})} \end{aligned}$$

For a chosen data set, suppose $\texttt {q}$ represents the total number of mapped proteins in BLAST. Hence the obtained normalized bitscore matrix called $\texttt {bit}\hbox {-} \texttt {matrix}[\texttt {q}][\texttt {q}]$ is of size ($\texttt {q}\times \texttt {q}$).

The protein sequence alignment-based similarity between protein $p_i$ and $p_j$ can be obtained from the generated bitscore matrix. The similarity between their corresponding genes $g_i$ and $g_j$ can be retrieved as follows.

7 $$\begin{aligned} \texttt {Seq}\hbox {-} \texttt {SIM}(g_i,g_j) = \texttt {bit}\hbox {-} \texttt {matrix}[g_i][g_j] \end{aligned}$$

The proposed integrated dissimilarity measure $\texttt {IntDis} (g_i, g_j)$ is formulated after combining Eqs. 3, 4, and 7 and then subtracting from 1 as follows.

8 $$\begin{aligned} \texttt {IntDis}(g_i, g_j) = 1 - \frac{\texttt {Multi}\hbox {-}{}\texttt{SIM}(g_i,g_j) + \texttt {PPI}\hbox {-}{}\texttt{SIM}(g_i, g_j) + \texttt {Seq}\hbox {-} \texttt {SIM}(g_i,g_j)}{3} \end{aligned}$$

where $$\texttt {IntDis}(g_i, g_j) \in [0,1]$$.

For *view-2*, dissimilarity matrix $\texttt {DView2}_{\texttt {n}\times \texttt {n}}$ is created by calculating pairwise functional dissimilarity between genes according to Eq. 8. Once both views, i.e., $\texttt {DView1}_{\texttt {n}\times \texttt {n}}$ and $\texttt {DView2}_{\texttt {n}\times \texttt {n}}$ are ready, they are next utilized by the proposed UMVMO-*select* algorithm for gene selection.

### Working methodology of UMVMO-*select*

The UMVMO-*select* algorithm comprises of nine essential steps, which are described with details in this section. The flowchart of the overall technique is shown in Fig. 6. Each step described below is also illustrated in the flowchart (Fig. 6) to make easy back-and-forth reference between the figure and the text.

**Fig. 5:**
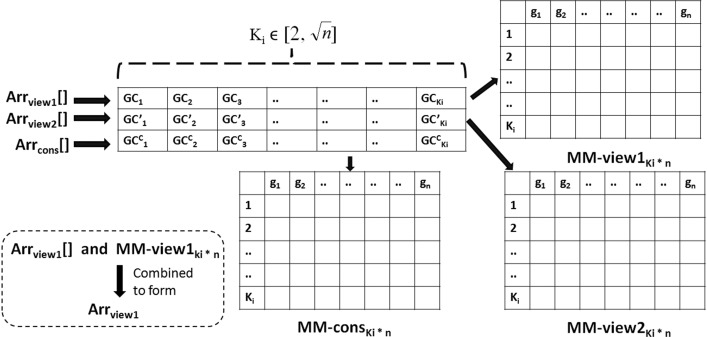
Structure of each clustering solution

**Fig. 6:**
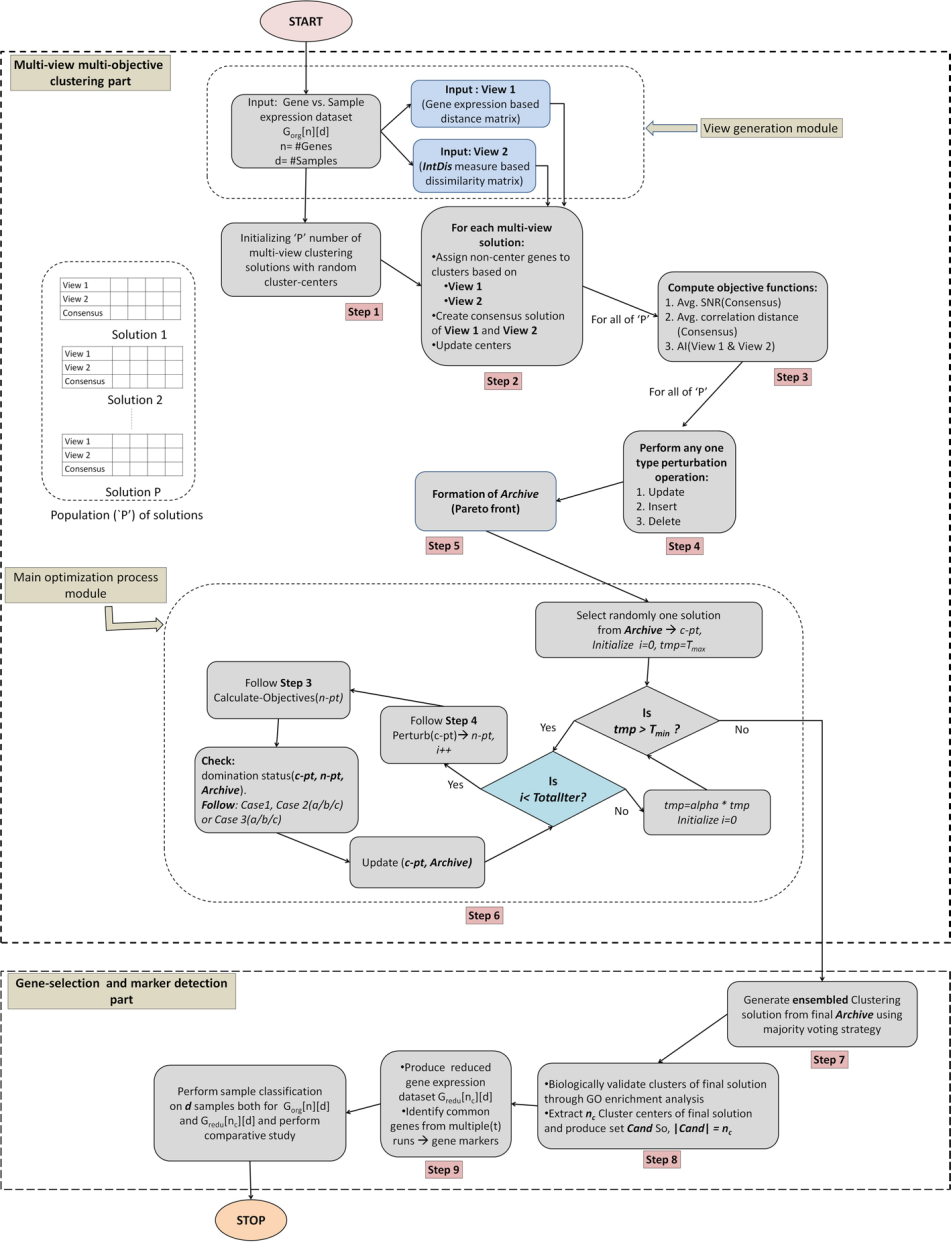
Working flow diagram of proposed UMVMO-*select* algorithm

*Step 1: Encoding scheme and initializing solutions*

1. *Structure of encoded solution* Our proposed algorithm starts with initializing ‘$\texttt {P}$’ number of random multi-view clustering solutions. Each clustering solution is represented as a structure of integer-encoded arrays and pointer to membership matrices. For better illustration, the structure of a complete multi-view clustering solution is shown in Fig. 5. We can see from the figure that both for *view-1* and *view-2*, two separate vectors of centers ($\texttt {Arr}_{\texttt {view1}}$[] and $\texttt {Arr}_{\texttt {view2}}$[]) within a multi-view solution are created. Each array/vector stores IDs of center genes of encoded clusters for the particular view. If we aim to perform clustering on $\texttt {n}$ genes of a chosen data set, then their positional indexes (like 1, 2, ...,$\texttt {n}$) are treated as their ID. Each vector has a pointer to a two-dimensional membership matrix (details later) to represent all non-center genes’ membership status in the corresponding clustering solution. The third vector, $\texttt {Arr}_{\texttt {cons}}$[], represents the array of centers from the consensus clustering solution (by combining $\texttt {Arr}_{\texttt {view1}}$ and $\texttt {Arr}_{\texttt {view1}}$). For simplification, throughout this article, we address $\texttt {Arr}_{\texttt {view1}}$[], $\texttt {Arr}_{\texttt {view2}}$[], and $\texttt {Arr}_{\texttt {cons}}$[] as vectors of cluster centers; and $\texttt {Arr}_{\texttt {view1}}$, $\texttt {Arr}_{\texttt {view2}}$, and $\texttt {Arr}_{\texttt {cons}}$ as clustering solutions correspond to the first view, the second view, and consensus of both views respectively (also mentioned in Fig. 5).
2. *Cluster center initialization* Let us assume, $K_i$ denotes the number of clusters/centers in $\texttt {Arr}_{\texttt {view1}}$, $\texttt {Arr}_{\texttt {view2}}$, and $\texttt {Arr}_{\texttt {cons}}$ of $i^{th}$ multi-view clustering solution. It can vary between the range of 2 to $\sqrt{\texttt {n}}$ [50] ($\texttt {n}$ is the number of data points participates in clustering). For $i^{th}$ solution, the values of $K_i$ in $\texttt {Arr}_{\texttt {view1}}$, $\texttt {Arr}_{\texttt {view2}}$, and $\texttt {Arr}_{\texttt {cons}}$ are the same, but it may be different for different solutions, i.e., $K_i \ne K_j$. For $i^{th}$ solution, $K_i$ is initialized as follows.
  9 $$\begin{aligned} K_i = \lbrace rand()\, \% \,(\sqrt{\texttt {n}} - 1)\rbrace + 2 \end{aligned}$$
  Here *i* $\in$ [$1, 2\ldots ,P$]. The execution of UMVMO-*select* starts with the initialization of both $\texttt {Arr}_{\texttt {view1}}$[] and $\texttt {Arr}_{\texttt {view2}}$[] independently with randomly selected $K_i$ gene IDs as centers from the original set of $\texttt {n}$ genes. $\texttt {Arr}_{\texttt {cons}}$[] of all ‘$\texttt {P}$’ solutions are initialized as a null array at the beginning of execution. Only once $\texttt {Arr}_{\texttt {view1}}$ and $\texttt {Arr}_{\texttt {view2}}$ are completely initialized, then $\texttt {Arr}_{\texttt {cons}}$ is updated (discussed in the next step of the algorithm). In Fig. 5, GC$_j$ and GC’$_j$ represent IDs of $j^{th}$ cluster center (where $j \in [1,\ldots ,K_i]$) correspond to $\texttt {Arr}_{\texttt {view1}}$ and $\texttt {Arr}_{\texttt {view2}}$, respectively. Also, GC$^c_j$ is the ID of $j^{th}$ cluster center of the consensus clustering solution $\texttt {Arr}_{\texttt {cons}}$. Please note that in our proposed algorithm, at any stage, cluster centers of any clustering solution must be the members of the original gene set ($\texttt {n}$ genes), i.e., basically, they are medoids.

*Step 2: Assigning non-center genes to clusters and creating consensus clustering solution*

Once $\texttt {Arr}_{\texttt {view1}}$[] and $\texttt {Arr}_{\texttt {view2}}$[] within each of ‘$\texttt {P}$’ solutions are initialized with random centers, the rest of the genes are assigned to their corresponding clusters for each view independently. This assignment follows the minimum-dissimilarity strategy between the gene to be assigned and encoded centers. For each view, the gene-gene dissimilarity matrix for the corresponding view ($\texttt {DView1}_{\texttt {n}\times \texttt {n}}$ or $\texttt {DView2}_{\texttt {n}\times \texttt {n}}$) is utilized for this purpose. As previously mentioned, three binary two-dimensional membership matrices ($\texttt {MM}\hbox {-}{} \texttt {view1}_{K_i \times \texttt {n}}$, $\texttt {MM}\hbox {-}{} \texttt {view2}_{K_i \times \texttt {n}}$, and $\texttt {MM}\hbox {-}{} \texttt {cons}_{K_i \times \texttt {n}}$) correspond to three center vectors are maintained, and they are shown in Fig. 5. The rows of the corresponding membership matrix represent the index of encoded clusters ($K_i$ clusters for $i^{th}$ solution), and columns represent $\texttt {n}$ genes’ IDs. The presence of a gene in a cluster is represented by 0 or 1 within the matrix. Once the assignment of non-center genes is done for both views, next, the existing cluster centers are updated with the IDs of most centrally located genes (gene having minimum average dissimilarity with other genes of the same cluster).

**Fig. 7:**
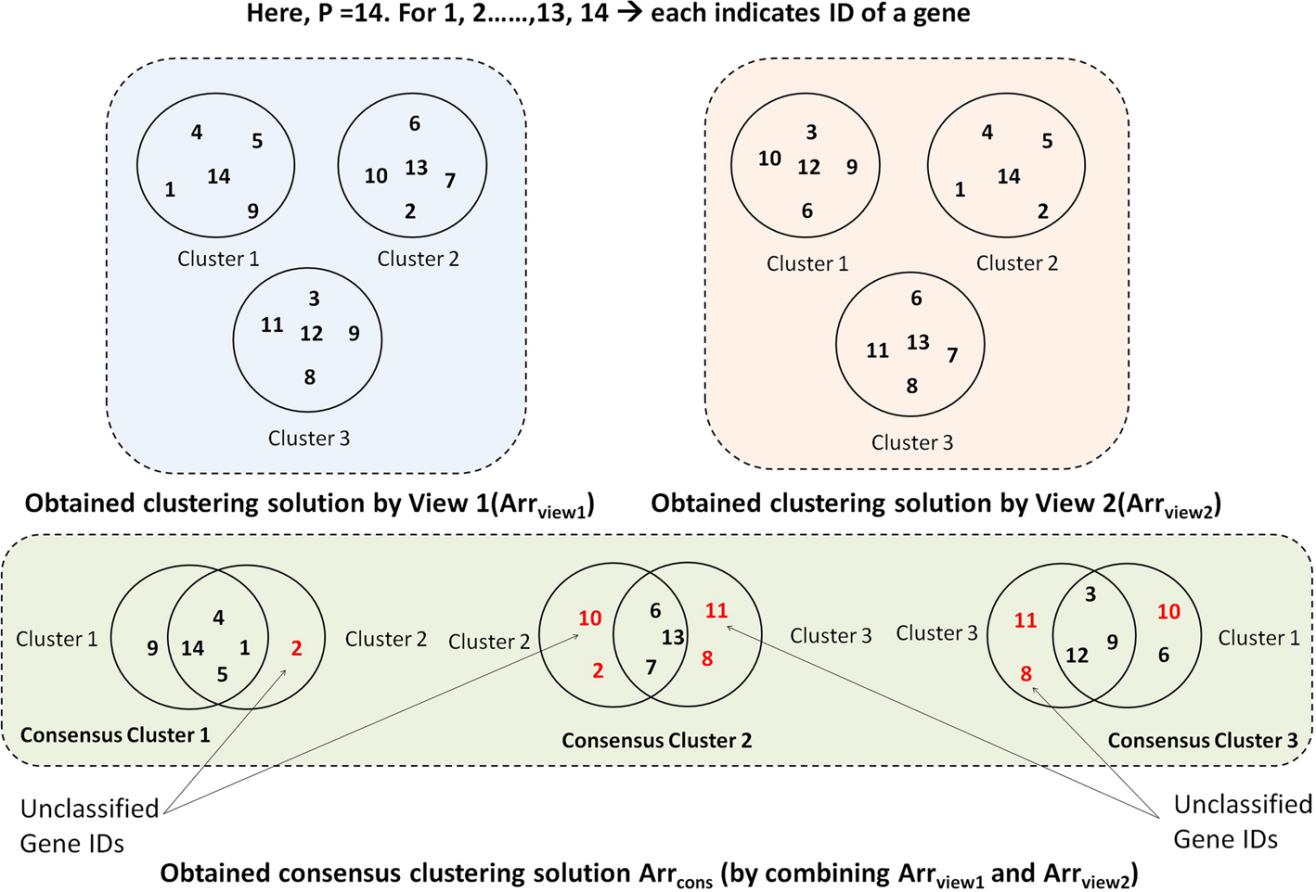
The scheme of forming consensus clusters in each solution

Next, the consensus partitions for ‘$\texttt {P}$’ solutions are created combining $\texttt {Arr}_{\texttt {view1}}$ and $\texttt {Arr}_{\texttt {view2}}$ and stored in $\texttt {Arr}_{\texttt {cons}}$. The procedure for the consensus of both views has been partially illustrated in Fig. 7. The three-step process of consensus generation is described as follows.

- *First step: Identifying overlapping clusters* Started with the first encoded cluster of $\texttt {Arr}_{\texttt {view1}}$ with all clusters of $\texttt {Arr}_{\texttt {view2}}$, one-to-one maximum overlapping cluster pairs are identified. The common genes from two overlapping clusters form a new consensus cluster. For example, according to Fig. 7, the overlapping clusters are as follows.
  - Cluster 1 of $\texttt {Arr}_{\texttt {view1}}$ *combines with* Cluster 2 of $\texttt {Arr}_{\texttt {view2}}$ *forms* Cluster 1 in $\texttt {Arr}_{\texttt {cons}}$.
  - Cluster 2 of $\texttt {Arr}_{\texttt {view1}}$ *combines with* Cluster 3 of $\texttt {Arr}_{\texttt {view2}}$ *forms* Cluster 2 in $\texttt {Arr}_{\texttt {cons}}$.
  - Cluster 3 of $\texttt {Arr}_{\texttt {view1}}$ *combines with* Cluster 1 of $\texttt {Arr}_{\texttt {view2}}$ *forms* Cluster 3 in $\texttt {Arr}_{\texttt {cons}}$.
- *Second step: Update consensus cluster centers* For each formed consensus cluster, the most centrally located gene among all members is chosen as the center. For example, in the consensus Cluster 1 of Fig. 7, among genes with ID 1, 4, 5, and 14, the one with minimum average pair-wise dissimilarity with other members (considering average from both $\texttt {DView1}_{\texttt {n}\times \texttt {n}}$ or $\texttt {DView2}_{\texttt {n}\times \texttt {n}}$) is chosen as the center of consensus Cluster 1. Similarly, centers of other consensus clusters are identified, and $\texttt {Arr}_{\texttt {cons}}$[] is formed.
- *Third step: Assignment of unallocated genes* The rest of the genes which are still unallocated get assigned to their corresponding consensus clusters having minimum-average-dissimilarity with centers. Following the same example in Fig. 7, we can see genes with IDs 2, 8, 10, 11 have not been categorized. So each of them is placed in any one of three formed consensus clusters of $\texttt {Arr}_{\texttt {cons}}$ accordingly. Accordingly, the corresponding membership matrix $\texttt {MM}\hbox {-}{} \texttt {cons}_{K_i \times \texttt {n}}$ is updated.

*Step 3: Calculating objective functions*

As UMVMO-*select* is designed as a multi-objective approach; therefore, it optimizes multiple objective functions at each iteration during its execution. The objective functions for our method have been chosen to maximize gene relevance and minimize gene redundancy. Mathematical descriptions of all three chosen objective functions are provided in detail as follows.

1. *Average signal-to-noise (SNR) ratio* [30]
  The samples of chosen gene expression data sets belong to either class 1 (let us denote it by $\texttt {CL1}$) or class 2 ($\texttt {CL2}$). Then the signal-to-noise ratio (SNR) of each gene $g_i$ (feature) is calculated using mean ($\texttt {MN}$) and standard deviation ($\texttt {SD}$) of $\texttt {CL1}$ and $\texttt {CL2}$, and it is defined as follows [30].
  10 $$\begin{aligned} \vert SNR_{g_r}\vert = \vert \frac{\texttt {MN}(g_r(\texttt {CL1})) - \texttt {MN}(g_r(\texttt {CL2}))}{\texttt {SD}(g_r(\texttt {CL1})) + \texttt {SD}(g_r(\texttt {CL2}))} \vert \end{aligned}$$
  Here $r \in \{1,\ldots ,\texttt {n}\}$. The $\texttt {MN}(g_r(\texttt {CL1}))$ and $\texttt {MN}(g_r(\texttt {CL2}))$ represent the mean of expression values of gene $g_r$ in $\texttt {CL1}$ and $\texttt {CL2}$, respectively. $\texttt {SD}(g_r(\texttt {CL1}))$ and $\texttt {SD}(g_r(\texttt {CL2}))$ represent the standard deviation of $g_r$ for $\texttt {CL1}$ and $\texttt {CL2}$, respectively. The SNR represents the ratio of relative mean to the sum of the standard deviation of two classes of samples. It indicates the difference between central tendency and dispersion or variation exists from the average value of data points (here features/genes). A lower value of SNR represents that the feature (here gene) does not have many different values in different classes. In contrast, high SNR value indicates that the feature values are spread out over an extensive range, and that means the values are different over classes. So, the SNR value represents the relevance of genes. For an ideal multi-view solution, the average SNR value of all encoded center genes within its consensus solution ($\texttt {Arr}_{\texttt {cons}}$) should be as high as possible.
  Please note that our proposed UMVMO-*select* is an unsupervised algorithm. Therefore, no labeled data can be utilized at any stage of it. However, for SNR calculation, it needs sample class information. To retrieve that, at the beginning (before the execution of UMVMO-*select* starts), we perform a basic unsupervised multi-objective clustering [15] on samples of chosen gene expression data sets, and hence the available samples are divided into two groups. The obtained group information is then utilized for the SNR calculation of all genes under experiment during UMVMO-*select*.
2. *Average pairwise correlation distance*
  The selected genes by proposed UMVMO-*select* aim not only to be relevant but non-redundant as well. To identify the set of non-redundant genes, a second objective function has been designed, i.e., average pairwise correlation distance [31] between centers. The correlation distance can be calculated according to Eq. 1 and can be obtained from the dissimilarity matrix of *view-1* or $\texttt {DView1}_{\texttt {n}\times \texttt {n}}$. For a better solution, the average pairwise correlation distance [31] between center genes of its consensus solution ($\texttt {Arr}_{\texttt {cons}}$) must be higher to ensure those centers are non-redundant to each other.
3. *Agreement Index (AI)*
  As the name implies, the objective function AI [32] quantifies the similarity between partitions of over $\texttt {n}$ genes obtained by *view-1* and *view-2* (i.e., $\texttt {Arr}_{\texttt {view1}}$ and $\texttt {Arr}_{\texttt {view2}}$). A higher value of AI signifies both views obtain more similar partitions.
  The formulation of AI for two-views, $\texttt {v1}$, and $\texttt {v2}$, is as follows. Suppose, $\texttt {A}^{\texttt {v1}}$ and $\texttt {A}^{\texttt {v2}}$ are the two agreement matrices corresponding to both views. The number of agreements ($\texttt {n}_\texttt {a}$) is calculated as follows: $\texttt {n}_\texttt {a}=\sum _{i=1}^{\texttt {n}}\sum _{j=1}^{\texttt {n}} \texttt {I}_{\texttt {A}_{\texttt {ij}}^{\texttt {v1}}, A_{ij}^{v2}}$, where
  $$\begin{aligned} \texttt {I}_{\texttt {A}_{\texttt {ij}}^{\texttt {v1}}, \texttt {A}_{\texttt {ij}}^{\texttt {v2}}}= & {} 1 \,\,\,\text{ if }\,\,\texttt {A}_{\texttt {ij}}^{\texttt {v1}}= \texttt {A}_{\texttt {ij}}^{\texttt {v2}}\\= & {} 0\,\,\,\,\text{ otherwise } \end{aligned}$$
  The number of disagreements ($\texttt {n}_\texttt {d}$) is calculated as follows: $\texttt {n}_\texttt {d}=\texttt {n}^2-\texttt {n}_a$ Hence the AI between these two views ($\texttt {v1}$, $\texttt {v2}$) is calculated as follows:
  $$\begin{aligned} \texttt {AI}_{\texttt {v1,v2}}=\frac{\texttt {n}_\texttt {a}+1}{\texttt {n}_\texttt {d} +1} \end{aligned}$$
  Here $\texttt {1}$ is used as a normalization factor to avoid *division by zero* problem.
  For more than two views the total *Agreement index* for the entire obtained partitioning is calculated as follows.
  $$\begin{aligned} \texttt {AI}=\frac{\sum _{i=1}^{m}\sum _{j=1, j\ne i}^m 2 \times \texttt {AI}_{\texttt {v}_i, \texttt {v}_j}}{\texttt {m}\times (\texttt {m}-1)}, \end{aligned}$$
  Here $\texttt {m}$ is the total number of views available. For an optimal multi-view clustering solution, a higher AI is expected.

For all of ‘$\texttt {P}$’ solutions, their corresponding three objective functions are calculated accordingly.

*Step 4: Perturbation operators*

**Fig. 8:**
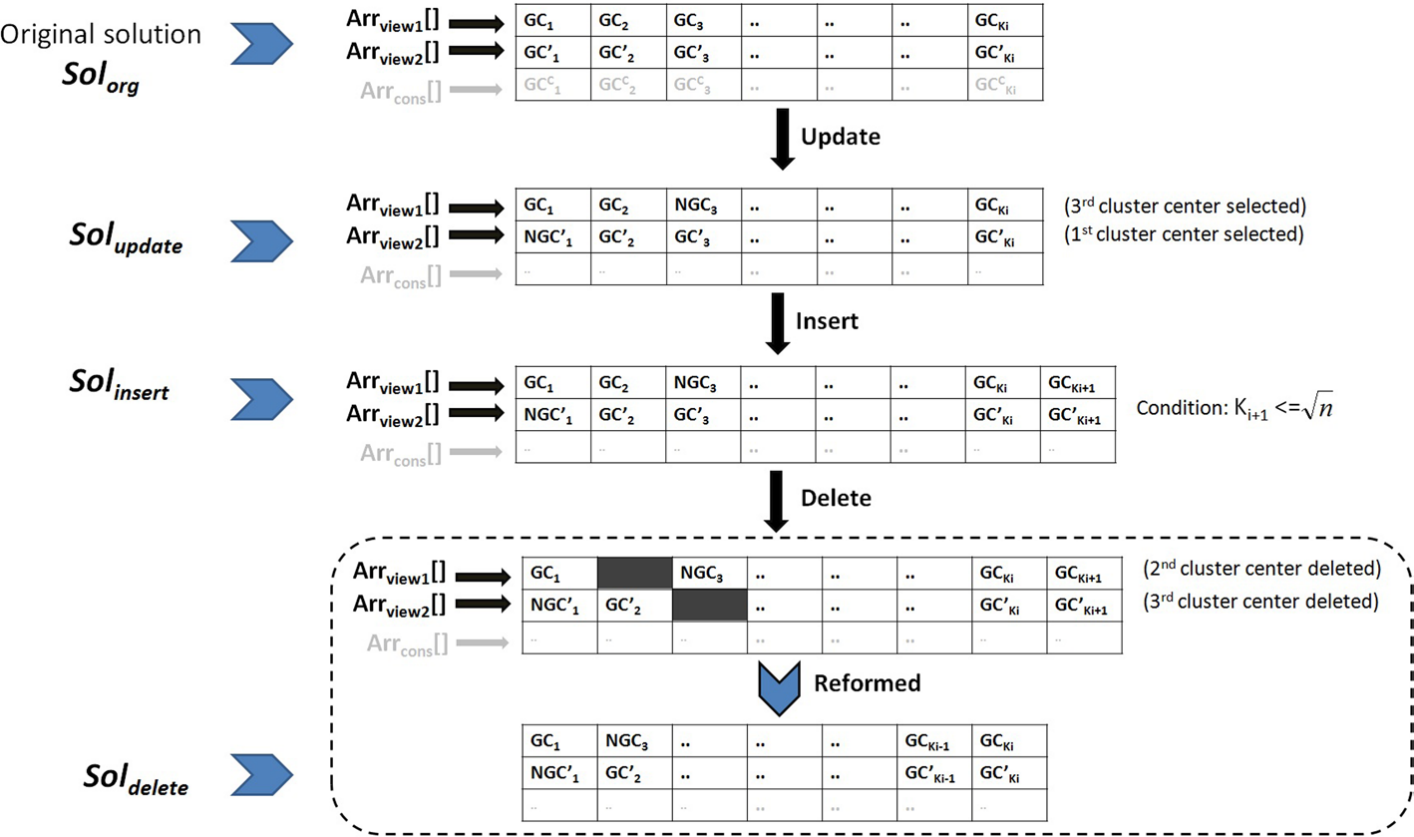
Three perturbation operations on a multi-view solution

Like most of the existing optimization techniques, to explore the search space properly, our proposed algorithm utilizes three types of perturbation operators (update, insert and delete) [15] applied on both $\texttt {Arr}_{\texttt {view1}}$ and $\texttt {Arr}_{\texttt {view2}}$ of each multi-view solution. Please note that consensus solution $\texttt {Arr}_{\texttt {cons}}$ does not directly perturb. However, they get updated after any change in $\texttt {Arr}_{\texttt {view1}}$ and $\texttt {Arr}_{\texttt {view2}}$. For every solution, the probability of choosing any one of three perturbation operators is equal, i.e., 0.33 each. Our proposed perturbation operations are inspired by Acharya et al. [15], though we have significantly modified them according to our algorithm’s requirement. The details of applied perturbation operations are provided as follows. They are also illustrated in Fig. 8.

- *Update* This is used to replace all existing centers of $\texttt {Arr}_{\texttt {view1}}$ and $\texttt {Arr}_{\texttt {view2}}$ with new non-center genes from the input gene set. Please note, this perturbation operation intends to impose a slight change in existing clusters. Hence, a gene having minimum dissimilarity with an existing center (utilizing $\texttt {DView1}_{\texttt {n}\times \texttt {n}}$ or $\texttt {DView2}_{\texttt {n}\times \texttt {n}}$) replaces it to become the new center. If a multi-view solution is chosen for perturbation type, then clustering solutions corresponding to both views go through it independently. For example, as illustrated in Fig. 8, a solution $\texttt {Sol}_{\texttt {org}}$ goes through this perturbation operation, and independently both $\texttt {Arr}_{\texttt {view1}}$[] and $\texttt {Arr}_{\texttt {view2}}$[] are perturbed. Suppose, from $\texttt {Arr}_{\texttt {view1}}$ and $\texttt {Arr}_{\texttt {view2}}$, $3^{rd}$ (GC$_3$) and $1^{st}$ (GC’$_1$) cluster are chosen respectively, and their existing centers are replaced by NGC$_3$ and NGC’$_1$.
- *Insert* This type of perturbation has been performed to increase the number of clusters by one within each of $\texttt {Arr}_{\texttt {view1}}$ and $\texttt {Arr}_{\texttt {view2}}$ of a multi-view solution. A random non-center gene is chosen from the input set each time and added to $\texttt {Arr}_{\texttt {view1}}$[] and $\texttt {Arr}_{\texttt {view2}}$[] independently. To apply this operator, the number of clusters ($K_i$) of a solution must be less than $\sqrt{\texttt {n}}$ so that after perturbation, $K_i$ does not exceed the permitted length ($\sqrt{\texttt {n}}$). If we continue the same example of Fig. 8, suppose the solution $\texttt {Sol}_{\texttt {update}}$ is selected for this operation. Then randomly, two non-center genes GC$_{K_{i+1}}$ and GC’$_{K_{i+1}}$ are chosen from the input gene set and added to respected vectors. However, before applying this operation, we need to make sure that $K_{i+1} \le \sqrt{\texttt {n}}$.
- *Delete* This perturbation type aims to decrease the number of clusters by one in each of $\texttt {Arr}_{\texttt {view1}}$ and $\texttt {Arr}_{\texttt {view2}}$. Randomly one existing center is chosen from $\texttt {Arr}_{\texttt {view1}}$[] and $\texttt {Arr}_{\texttt {view2}}$[] independently and deleted from the array. To apply this operation, the number of clusters ($K_i$) of a solution must have a minimum length of 3 so that after perturbation, $K_i$ is not decreased below the permitted length (i.e., 2). In Fig. 8, $\texttt {Sol}_{\texttt {insert}}$ is chosen for this operation. Existing gene centers from $2^{nd}$ and $3^{rd}$ clusters of $\texttt {Arr}_{\texttt {view1}}$[] and $\texttt {Arr}_{\texttt {view2}}$[], respectively (GC$_2$ and GC’$_3$), are chosen randomly and omitted. As a result, the new solution $\texttt {Sol}_{\texttt {delete}}$ is formed.

After following any one of the above-mentioned types of perturbation operation, all non-center genes’ membership status is recalculated; hence membership matrices for both views ($\texttt {MM}\hbox {-}{} \texttt {view1}_{K_i \times \texttt {n}}$ and $\texttt {MM}\hbox {-}{} \texttt {view2}_{K_i \times \texttt {n}}$) are updated. The corresponding consensus clustering solution ($\texttt {Arr}_{\texttt {cons}}$) is also updated accordingly.

*Step 5: Forming non-dominating* ***Archive***

Once all of ‘$\texttt {P}$’ solutions are completely initialized according to *Step 1* and *Step 2*, then according to *Step 3*, all three objective functions are calculated for them. Next, these solutions are perturbed once (according to *Step 4*). Similar to original solutions, objective functions are calculated for their corresponding perturbed solutions too. In this step, UMVMO-*select* aims to identify non-dominated solutions. According to the concept of underlying optimization strategy AMOSA [15], to store non-dominated solutions obtained so far, an *Archive* is maintained. These non-dominated solutions are also called Pareto-optimal solutions, and the front made by them is also known as Pareto-front [51].

Before forming *Archive*, the domination status between ‘$\texttt {P}$’ number of original and corresponding perturbed solutions is checked. If a perturbed solution dominates the original one, then the original solution is replaced by the perturbed one; otherwise, the original is kept. If both solutions are non-dominating then also the original stays unchanged. Next, the *Archive* made of non-dominated solutions (out of ‘$\texttt {P}$’ solutions) is identified. Any two clustering solutions are called non-dominating if both of them dominates each other with respect to at least one objective function value. These solutions are identified and added to the *Archive*. It follows two size limits; soft limit ($\texttt {SL}$) and hard limit ($\texttt {HL}$). Generally, $\texttt {SL} > \texttt {HL}$. During the entire optimization process, non-dominated solutions are stored in the *Archive* up to the limit of $\texttt {SL}$. Once the number of solutions crosses $\texttt {SL}$, a single linkage-based clustering is applied to reduce the size of *Archive* to $\texttt {HL}$.

*Step 6: The main optimization process*

**Fig. 9:**
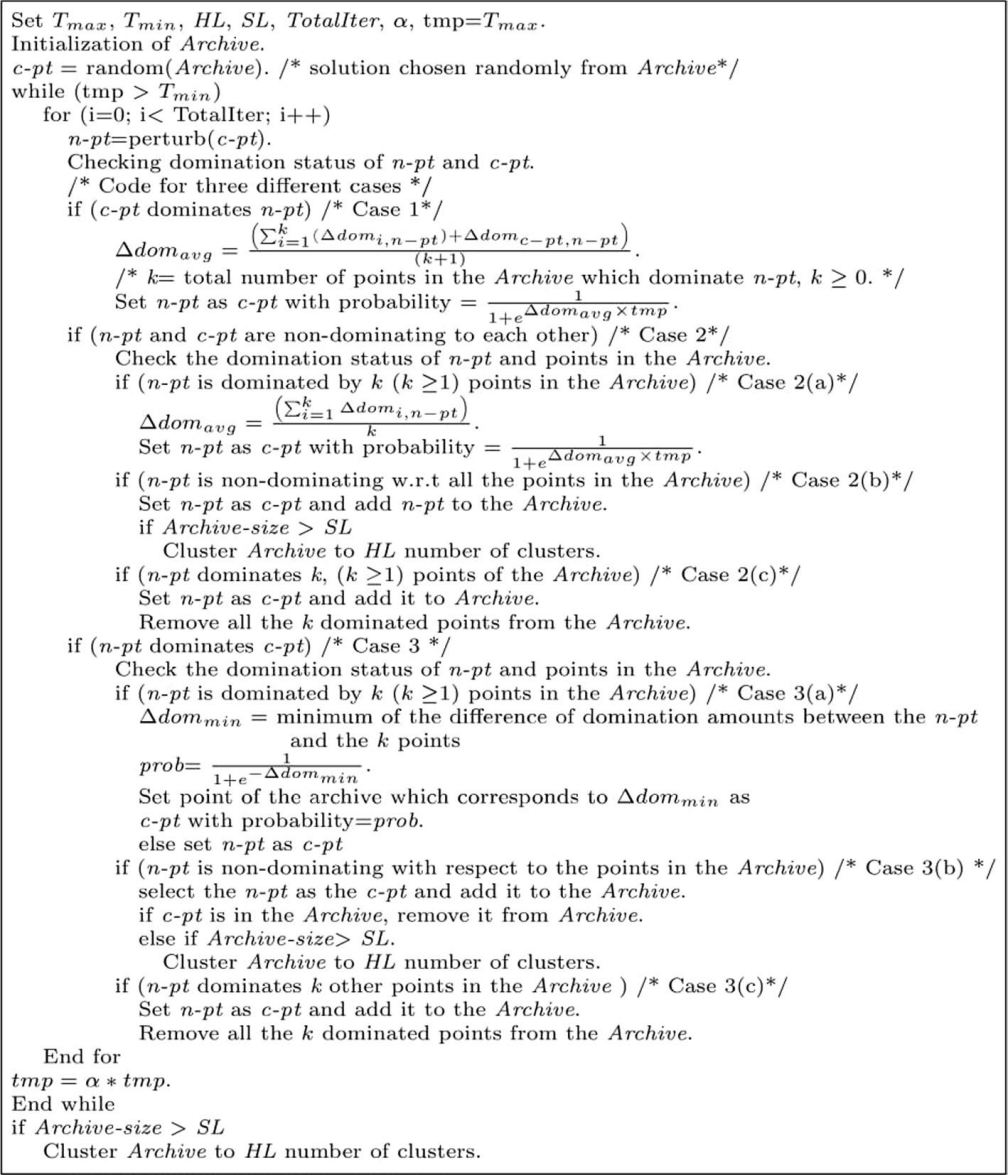
Pseudo-code of AMOSA algorithm

At this step, our proposed algorithm follows the optimization strategy of AMOSA [15]. According to this optimization strategy, a variable $\texttt {tmp}$ is initiated with a maximum temperature parameter $\texttt {T}_{\texttt {max}}$. $\alpha$ is a fixed parameter denoting the cooling rate. $\texttt {tmp}$ gets decreased gradually from $\texttt {T}_{\texttt {max}}$ with cooling rate $\alpha$ until $\alpha \le \texttt {T}_{\texttt {min}}$. $\texttt {T}_{\texttt {min}}$ is the lowest temperature variable. At each value of $\texttt {tmp}$, several times ($\texttt {TotalIter}$), also referred to as *generations*, the main optimization process is executed. A single solution is picked up randomly from the *Archive*; let us denote it as the current point or c-pt. To generate a new solution—*n-pt*, any one type of perturbation operations is performed on the *c-pt* (according to *Step 4*). After that, objective function values of *n-pt* are calculated (according to *Step 3*), and domination status between *c-pt* and *n-pt* along with rest solutions in *Archive* is checked. According to [15], the amount of domination $\Delta \texttt {dom}(a,b)$ between two solutions *a* and *b* is defined as follows.

11 $$\begin{aligned} \Delta \texttt {dom}_{a,b}=\prod _{i=1,f_{i}(a)\ne f_{i}(b)}^{\texttt {M}_{\texttt {obj}}}\frac{|{f_{i}(a)-f_{i}(b)}|}{R_i} \end{aligned}$$

where $f_{i}(a)$ and $f_{i}(b)$ are the $i^{th}$ objective values of the two solutions. The range of the $i^{th}$ objective is denoted by $R_{i}$ and $\texttt {M}_{\texttt {obj}}$ denotes the number of objective functions. To calculate $R_{i}$, the solutions present in the *Archive* along with *c-pt* and the *n-pt* are used. Next, based on the domination status of the *n-pt* and the *c-pt* along with *Archive* member, three different cases can arise. Based on these cases, different strategies are adopted to update *c-pt* and *Archive* members. The overall pseudo-code of the optimization strategy (AMOSA), followed by our proposed algorithm, is shown in Fig. 9. Also, please see the main optimization process module from the flowchart, as shown in Fig. 6. Finally, once the temperature variable ($\texttt {tmp}$) drops into or below $\texttt {T}_{\texttt {min}}$, the obtained *Archive* is considered as the final optimized set containing non-dominated solutions or the final Pareto-front.

*Step 7: Ensemble operation on the final Pareto front*

From the obtained Pareto-front, any solution can be chosen as a final multi-view clustering solution according to any cluster quality metric [3]. However, our proposed UMVMO-*select* performs an ensemble operation on consensus solutions ($\texttt {Arr}_{\texttt {cons}}$) obtained from the *Archive* following the majority voting strategy. If a pair of genes are members of the same cluster for the majority of produced consensus clustering solutions, then they are kept together in the final solution. Following this rule, the grouping is done for most of the input genes. The genes which can not be grouped in this manner follow a similar strategy as followed in *Step 2*, i.e., minimum-average-dissimilarity with centers utilizing both views.

The final ensembled consensus clustering solution is further utilized to generate a set of non-redundant and relevant candidate genes.

*Step 8: Validate the final ensembled consensus clustering solution and extract centers as candidate genes*

Once the final ensembled clustering solution is obtained, we perform a biological significance test on the obtained clustering solution before extracting the candidate genes. The GO enrichment analysis—presented by the GO tool^5^ is conducted for this purpose, described in detail in the “Discussion” section. If any cluster(s) from the obtained solution fails the validation test, then the following tests must be performed until the final solution is found as valid.

- Individually perform biological significance test on each consensus solution of Pareto front. The solution(s) who fails in the validation test is discarded. Rest consensus solutions are ensembled to produce the final solution, according to *Step 7*.
- If all Pareto front solutions are biologically significant, but not the ensembled one then, the consensus solution having maximum Silhouette index value [35] is chosen for candidate gene selection instead of the ensembled one.
- If neither ensembled nor any Pareto front solutions are biologically significant, then the input parameters are changed through the sensitivity analysis (discussed in the “Results” section) to re-execute UMVMO-*select*.

After validating the obtained solution, the encoded cluster centers are extracted as candidate genes (features) of reduced gene-space. Suppose $\texttt {Cand}$ represents this set of candidate genes. Let, $\vert \texttt {Cand} \vert =\texttt {n}_\texttt {c}$, which represents $\texttt {n}_\texttt {c}$ number of genes, are selected as candidate genes. Here, $\texttt {n}_\texttt {c} < \texttt {n}$ and also $\texttt {n}_\texttt {c} = \#$ clusters in the final solution. Finally, from the original expression data set $\texttt {G}_{\texttt {org}}[\texttt {n}][\texttt {d}]$, rows corresponding to $\texttt {n}_\texttt {c}$ candidate genes are extracted, and gene expression data set $\texttt {G}_{\texttt {redu}}[\texttt {n}_\texttt {c}][\texttt {d}]$ with reduced gene space $\texttt {n}_\texttt {c}$ is formed.

*Step 9: Identifying gene markers from multiple runs of proposed algorithm*

We execute the proposed algorithm UMVMO-*select* ‘$\texttt {t}$’ number of times on a particular data set, which forms ‘$\texttt {t}$’ different sets of candidate genes. To ensure the stability, the genes those appear in each of ‘$\texttt {t}$’ obtained sets are chosen and reported as gene markers in this article. To decide the best value of ‘$\texttt {t}$’ for each data set, it is increased up to certain trials, after which the set of obtained common gene markers does not change anymore.

## Data Availability

All data sets used in the work are publicly available and the source references are given in main manuscript.

## Abbreviations

UMVMO-*select*: Unsupervised Multi-View Multi-Objective clustering-based gene selection
UMC: Unsupervised multi-objective clustering
GO: Gene ontology
BP: Biological process
CC: Cellular component
MF: Molecular function
PPIN: Protein–protein interaction network
MRMR: Minimum redundancy maximum relevance
CLARANS: Clustering Large Applications based upon RANdomized Search
DLBCL: Diffuse large B-cell lymphomas
BLAST: Basic local alignment search tool
AMOSA: Archived multi objective simulated annealing
CA: Classification accuracy
DB index: Davies Bouldin index
MPSO: Multi-objective particle swarm optimization

## Footnotes

1 www.biolab.si/supp/bi-cancer/projections/info/.

2 http://geneontology.org/.

3 https://blast.ncbi.nlm.nih.gov/Blast.cgi.

4 https://blast.ncbi.nlm.nih.gov/Blast.cgi.

5 http://geneontology.org/.
